# Discordance between Primary Breast Cancer and Ipsilateral Breast Cancer Tumor Recurrence as a Function of Distance

**DOI:** 10.3390/jcm9124033

**Published:** 2020-12-13

**Authors:** Sebastian M. Jud, Reinhard Hatko, Julius Emons, Bianca Lauterbach, Carolin C. Hack, Caroline Preuß, Werner Adler, Matthias W. Beckmann, Felix Heindl

**Affiliations:** 1 Department of Gynecology and Obstetrics, Erlangen University Hospital, Comprehensive Cancer Center, European Metropolitan Area Erlangen-Nuremberg (CCC ER-EMN), Friedrich Alexander University of Erlangen-Nuremberg, 91054 Erlangen, Germany; julius.emons@uk-erlangen.de (J.E.); Bianca.Lauterbach@gmx.de (B.L.); carolin.hack@uk-erlangen.de (C.C.H.); caroline.preuss@uk-erlangen.de (C.P.); fk-direktion@uk-erlangen.de (M.W.B.); felix.heindl@uk-erlangen.de (F.H.); 2 Freelance Computer Scientist, 85049 Ingolstadt, Germany; reinhard@hatko.de; 3 Department of Biometry and Epidemiology, Friedrich Alexander University of Erlangen-Nuremberg, 91054 Erlangen, Germany; werner.adler@fau.de

**Keywords:** breast cancer, ipsilateral recurrence, prognosis, tumor characteristics

## Abstract

Background: Risk factors for ipsilateral breast cancer tumor recurrence (IBTR) are well established and include grading, nodal status, and receptor status. Little is known about the influence of the local distance between the primary tumor and recurrences on changes in tumor characteristics and prognosis. Methods: In a retrospective setting, we analyzed primary breast cancers and their recurrences. Localizations of primary and recurrent breast cancer were recorded to calculate the relative distance in pixels. Analysis was performed regarding tumor characteristics, relative distance between both, and their impact on breast cancer prognosis. Results: In a cohort of 142 patients with ipsilateral recurrence, no statistically significant difference could be shown in the change in tumor characteristics depending on distance. Progesterone receptor (PR) and estrogene receptor (ER) status changed in 22.7% and 14.9% of cases, respectively. human epidermal growth factor receptor 2 (ERBB2, HER2) status changed in 18.3% of cases. Survival was in accordance with the literature, with luminal-A-like tumors as best and triple negative breast cancers (TNBC) as worst prognosis. With a threshold of 162 pixels, the survival was significantly better in the group with shorter distance. Conclusion: Change in tumor characteristics from primary breast cancer to recurrence occurs more often in PR than ER. In contrast to other work, in this dataset, recurrences with a larger distance to the primary tumor had a worse prognosis in univariate analysis. A Cox model might indicate the possibility that this influence is independent of other risk factors.

## 1. Introduction

About 10% of patients with breast cancer will develop isolated breast cancer recurrence after adjuvant treatment [1,2,3].

The main risk factors for developing ipsilateral breast cancer tumor recurrence (IBTR) or loco-regional recurrence (LRR) have been well examined, and include tumor size, tumor grading, lymph node status, and negative hormone receptor status [2,4,5,6,7,8].

The biomarker profile, especially hormone receptor status and (HER2) status, of primary breast cancers and breast cancer recurrences is essential for treatment decision-making [2,9,10,11,12,13]. It is known that a change in biomarker profile can occur from primary breast cancer to breast cancer recurrences or metastases [14]. Several studies showed that biomarkers change between primary breast cancer and ipsilateral breast cancer tumor recurrence [15]. Typical rates for estrogen receptor (ER), progesterone receptor (PR), and HER2 change are 20%, 33%, and 8%, respectively [3,16].

The definition of, and differentiation between, IBTR and secondary breast tumors is still an unsolved problem.

Several definitions of IBTR exist, including “recurrent disease in the ipsilateral breast” [17], “any recurrence in the ipsilateral breast with no association to distance” [18], and “recurrence within 3 cm of primary site and same immunohistochemical profile” [11,19,20].

In the German guidelines for the treatment of breast cancer, LRR is defined as “the recurrence of the breast carcinoma in the ipsilateral breast, ipsilateral chest wall including skin, the regional lymph nodes in the axilla, the supra- and infra-clavicular as well as the internal mammary area” [2]. One of the most cited trials of breast cancer recurrences in recent years was the CALOR trial [21], evaluating chemotherapy for locoregional recurrences. The definition and inclusion criteria in this trial were: soft tissue recurrence in the same breast after breast conserving therapy, or recurrence on the chest wall in the scar or skin after mastectomy [21]. The change in biomarker profile was not included in this definition of IBTR.

The factors that define prognosis after IBTR are reported in a number of studies. Higher age, smaller tumor size, negative lymph node status, and longer time interval between primary diagnosis and IBTR are favorable factors for prognosis [3,22,23,24,25].

Little is known about the prognosis associated with the local distance between the primary breast cancer site and the site of IBTR. One study showed a favorable prognosis with a greater distance between the primary tumor and recurrence sites. In this study, IBTR was separated into true recurrences or new primaries depending on distance and biomarker profile [19]. There is a lack of knowledge regarding the change in biomarkers relative to the local distance between the primary breast cancer site and the site of breast cancer recurrence.

Due to the differing prognoses of IBTR and the secondary tumor, this discrimination is essential for treatment decisions, and for the patients themselves.

Therefore, the aim of this study was to evaluate the correlation between change of tumor characteristics and local distance between primary tumor and recurrence sites, and to examine whether there is an association between local distance and prognosis.

## 2. Experimental Section

### 2.1. Patients

This is a retrospective study including patients who were newly diagnosed with breast cancer at the University Breast Center of Franconia, Germany, between 1995 and 2010. All breast cancer patients were screened, and patients with ipsilateral breast cancer tumor recurrence were identified (*n* = 364). For this study, patients were excluded for the following reasons: missing data on immunohistochemistry at primary diagnosis or recurrence, missing imaging data, or patients who did not have salvage surgery.

Finally, a complete dataset of 142 patients with ipsilateral breast cancer recurrence was obtained, with ER and/or PR status available from both primary diagnosis and recurrence. Additionally, both Ki-67 and HER2 status was reported at primary diagnosis and recurrence, wherever available. The study was in accordance with the Declaration of Helsinki and local ethical guidelines.

### 2.2. Data Collection

The University Breast Center of Franconia received certification from the German Cancer Society (Deutsche Krebsgesellschaft) and the German Society for the Study of Breast Diseases (Deutsche Gesellschaft für Senologie) in 2004. To obtain certification, a breast center has to document each case of breast cancer, including patient and tumor characteristics, treatment data, and some epidemiological data into a prospective database. Follow-up information regarding local recurrences, distant metastases, and death has to be provided for up to 10 years after the initial diagnosis. Follow-up was done according to local guidelines with mammography and breast ultrasound every 6 to 12 months. All histopathological data also have to be documented from the original pathological reports, including tumor size, axillary lymph node status, grading, and ER, PR, and HER2 status. Breast centers and their data quality are audited annually as part of the continuous certification process. Data obtained through these processes were used in the analysis presented here.

### 2.3. Histopathological Assessment

All of the histopathological information used in this analysis was directly documented from the original pathology reports, which were reviewed by two investigators. Grading, tumor type, ER status, PR status, HER2 status, and proliferation status (as assessed by Ki-67 staining) have been routinely recorded at the breast center since 1995, and were performed on formalin-fixed, paraffin-embedded tumor tissue. Monoclonal mouse antibodies against estrogen receptor-alpha (clone 1D5; 1:200 dilution, DAKO, Glostrup, Denmark), monoclonal mouse antibodies against the progesterone receptor (clone pgR636, 1:200 dilution, DAKO, Glostrup, Denmark), and monoclonal mouse antibodies against Ki-67 (clone MIB-1, 1:200 dilution, DAKO, Glostrup, Denmark) were used to stain the primary tumors and recurrences. The percentage of positively stained cells was included in the pathology reports. The tumors were considered to be positive for the estrogen and progesterone receptors if 1% or more of the cells showed positive staining.

A polyclonal antibody against HER2/neu (A0485, 1:200 dilution, DAKO, Glostrup, Denmark) was used, and HER2 status was stated in the pathology reports as negative, 0+, 1+, 2+, or 3+. As for a large proportion of recurrences, FISH or CISH testing was not available, so the use of those data was not taken into consideration to further adjust the HER2 status. Scoring was carried out in a standardized way by a group of dedicated pathologists in routine surgical pathology. With regard to Ki-67, areas with the highest Ki-67 labeling were investigated, and approximately 500 cells were counted with 400-fold magnification.

### 2.4. Documentation of Localization

The positions of the primary breast cancers and the recurrences were located with a mammogram, breast ultrasound, and palpation, and were documented in the patient chart.

**Figure 1:**
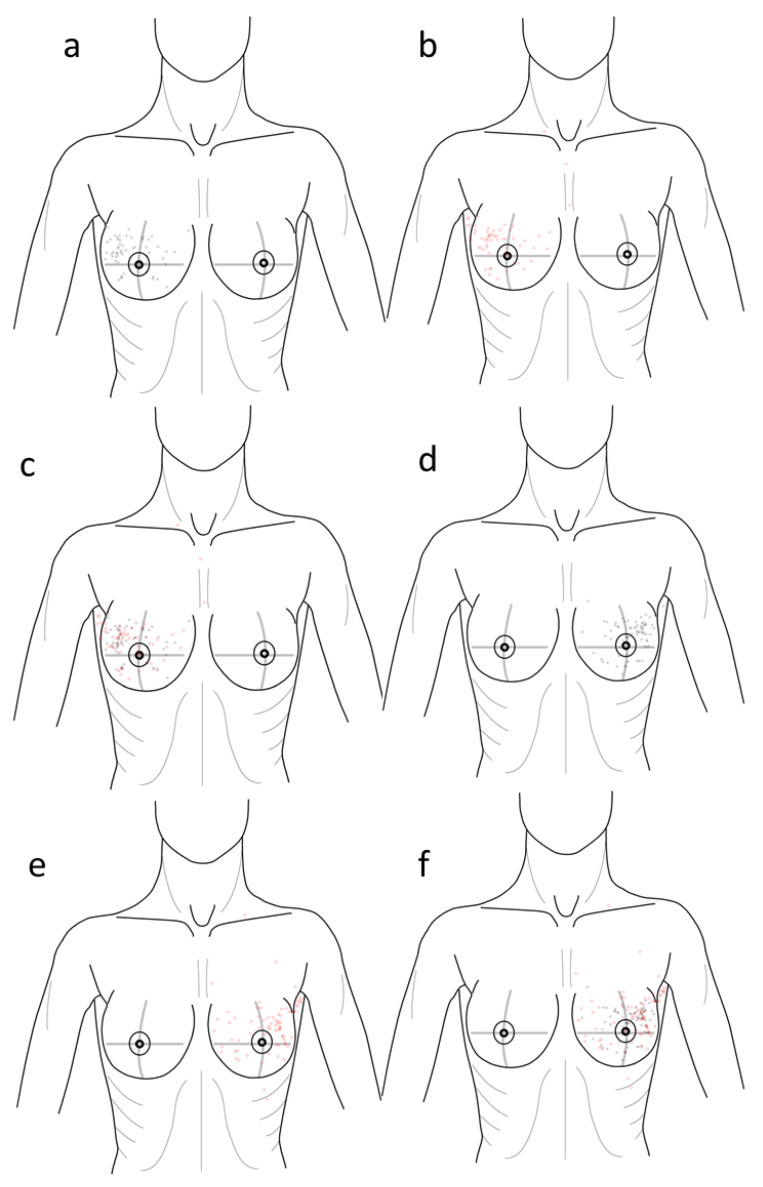
Distribution of (**a**) right primary breast cancer cases (**b**) site of recurrences and (**c**) both, (**d**–**f**) cases on the left side.

Using an Intuos3 professional pen tablet (Wacom Europe Ltd., Krefeld, Germany) and the ImageJ picture editing program (Research Services Branch, National Institute of Mental Health, Bethesda, MA, USA), a standard picture of the upper female body was adjusted, and the coordinates of the localization of the primary tumor were marked and noted. The coordinates of the recurrence site were then marked and noted. This resulted in two coordinates (x, y) of each site. To ensure consistency in the digitizing procedure, the position of the pictograms on the pen tablet was standardized (Figure 1).

### 2.5. Statistical Considerations

Overall survival (OS) was defined as the time from the date of primary breast cancer diagnosis to either the date of death or the date of censoring. Additionally, we examined survival after recurrence when local distance between primary diagnosis and recurrence was considered. Patients who were lost to follow-up within 20 years were censored at the last date they were known to be alive. For example, a patient who was alive 20 years after diagnosis would have been censored at that point.

Survival rates were estimated by the Kaplan–Meier product limit method and illustrated by Kaplan–Meier curves. Differences of survival between groups were tested with log-rank tests.

A Cox regression model was used to examine the relationship between survival and the local distance correcting for the influence of several tumor characteristics, age and time to recurrence. To identify the most appropriate model, stepwise variable selection was performed.

Group comparisons unrelated to survival were done with the Kruskal–Wallis test and the Mann–Whitney U test, respectively. To account for multiple testing, Benjamini–Hochberg correction was performed where appropriate.

All of the tests were two-sided, and a *p* value of ≤0.05 was regarded as statistically significant. Calculations were carried out using the SPSS software package (SPSS Statistics for Windows, version 24, IBM Corporation, Armonk, New York, NY, USA) and the statistical programming language R V3.6.3 [26].

## 3. Results

### 3.1. Subsection

#### 3.1.1. Patient Characteristics

The final cohort consisted of 142 patients. The average age at primary diagnosis was 55.01 years. The average age at menarche was 13.66 years. Overall, 106 stated that they had never used hormone replacement therapy (HRT), and 22 had used HRT at some point. All patients were treated according to the German guidelines for endocrine therapy, chemotherapy, trastuzumab, or radiation therapy depending on the subtype of breast cancer recurrence. Only patients with negative resection margins after the surgery for primary breast cancer were included in this analysis. The median follow-up time was 158 months.

**Figure 2:**
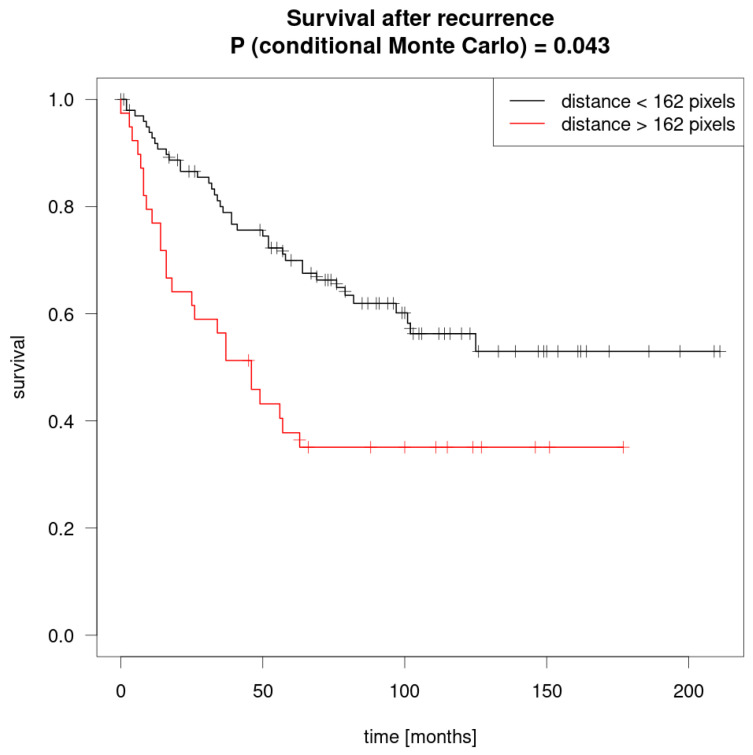
Kaplan–Meier curve for mean survival (months) by distance.

Further patient characteristics can be seen in Figure 2 in the Kaplan–Meier curve for mean survival (months) by distance (Table 1).

#### 3.1.2. Tumor Characteristics

In 44.4% of cases, the recurrence was located in another quadrant to the primary tumor of the same breast. In 55.6%, the recurrence was in the same quadrant as the primary carcinoma.

Overall, 54.9% of the primary tumors were located in the upper outer quadrant, 23.9% were in the upper inner quadrant, and 7.7% and 13.4% were in the lower outer and lower inner quadrants, respectively.

In addition, 8.5% of the tumors at primary diagnosis were well differentiated, 54.2% were moderately differentiated, and 32.4% were poorly differentiated (Table 2).

Tumors were categorized in four subtypes by hormone receptor status, grading, HER2-status and proliferation rate (Ki-67): luminal-A-like (ER positive and/or PR positive, Ki-67 < 14 %, G1/2 and HER2/neu negative), luminal-B-like (ER positive and/or PR positive, Ki-67 > 14 %, G2/3), HER2-enriched (any ER/PR status, any Ki-67, any G, HER2/neu positive) and triple negative (TNBC; ER and PR negative, HER2/neu negative, any Ki-67, any G).

The tumor subtype at primary diagnosis was luminal-A-like in 21.1% of cases, 44.4% were luminal-B-like, 14.1% were HER2-enriched, and 19.7% were triple negative (TNBC). At the time of recurrence, these figures were 21.8%, 36.6%, 13.4%, and 26.8%, respectively. Tumor characteristics can be seen in Table 2.

#### 3.1.3. Characteristics of Recurrence

The median time to recurrence was 33.5 months (mean: 43.06 months).

With regard to tumor subtype at primary diagnosis, the time to recurrence was 55.27 months for luminal-A-like tumors, 45.84 months for luminal-B-like tumors, 35.19 months for HER2-enriched tumors, and 29.61 months for TNBC. A Kruskal–Wallis test revealed that these differences were significant (*p* = 0.009; Benjamini–Hochberg corrected *p* = 0.038). The mean local distance between the primary tumor and the site of recurrence (in pixels) was 124 in luminal-A-like, 126 in luminal-B-like, 176 in HER2-enriched tumors, and 137 in TNBC. Regarding local distance, there was no statistical significance between the tumor types (Kruskal–Wallis test: *p* = 0.449) (Table 3).

Regarding the tumor subtype at recurrence, the mean distance between the site of primary diagnosis and site of recurrence (in pixels) was 86 for luminal-A-like recurrences, 144 for luminal-B-like recurrences, 201 for HER2-enriched recurrences, and 138 for TNBC. These distances did not differ significantly (Kruskal–Wallis test: *p* = 0.067) (Table 4).

#### 3.1.4. Grading and Change in Grading between Primary Diagnosis and IBTR with Regard to Distance

The mean local distance between primary site and recurrence for G1, G2, or G3 primary tumors corresponded to 101 pixels, 133 pixels, and 149 pixels, respectively. Regarding the grading at recurrence, the distances were 99 pixels, 137 pixels, and 134 pixels, respectively. The distances did not differ significantly between groups with different changing behavior of grading from the primary site to recurrence: (a) primary G1/2 →≥ recurrenceG1/2; (b) primary G1/2 → recurrence G3; (c) primary G3 → recurrence G1/2; (d) primary G3 → recurrence G3 (Kruskal–Wallis test: *p* = 0.912).

#### 3.1.5. Change of Tumor Characteristics

The ER status changed from positive to negative in 9.2% of the cases, and from negative to positive in 5.7% of the cases, between primary breast cancer diagnosis and breast cancer recurrence.

Regarding the four possible groups (negative to negative, negative to positive, positive to negative, and positive to positive), the mean distances were 148, 107, 131, and 131 pixels, respectively. No significant difference could be shown (Kruskal–Wallis test: *p* = 0.789).

A change in the PR status from positive to negative occurred in 14.2% of cases, and from negative to positive in 8.5% of cases. In addition, no significance could be shown in the distances (*p* = 0.103).

HER2 status changed from positive to negative in 8.1% of cases, and from negative to positive in 10.2% of cases. There was no significance regarding distance (*p* = 0.652).

#### 3.1.6. Distance between Primary Site and Recurrence Depending on Nodal Status and Tumor Size

The mean distance between primary diagnosis and IBTR was 111 pixels for nodal negative patients, and 166 pixels for nodal positive patients. The Mann–Whitney U-test revealed a *p*-value of 0.034, but after Benjamini–Hochberg correction for multiple testing, no significant difference could be shown (*p* = 0.084 after correction).

The mean distance for smaller tumors (T0–2) was 128 pixels, whereas the mean distance for larger tumors (T3+4) was 215 pixels. A significant difference can be seen between these two groups (*p* = 0.005; Benjamini–Hochberg corrected *p* = 0.038)

#### 3.1.7. Age, BMI, and Time to Recurrence in Relation to Distance of Recurrence

No significant relationships between distance and age, BMI, or time to recurrence were observed by means of correlation analysis after a Spearman test (*p* = 0.512; *p* = 0.772; *p* = 0.592).

#### 3.1.8. Survival by Tumor Subtype at Primary Diagnosis

**Figure 3:**
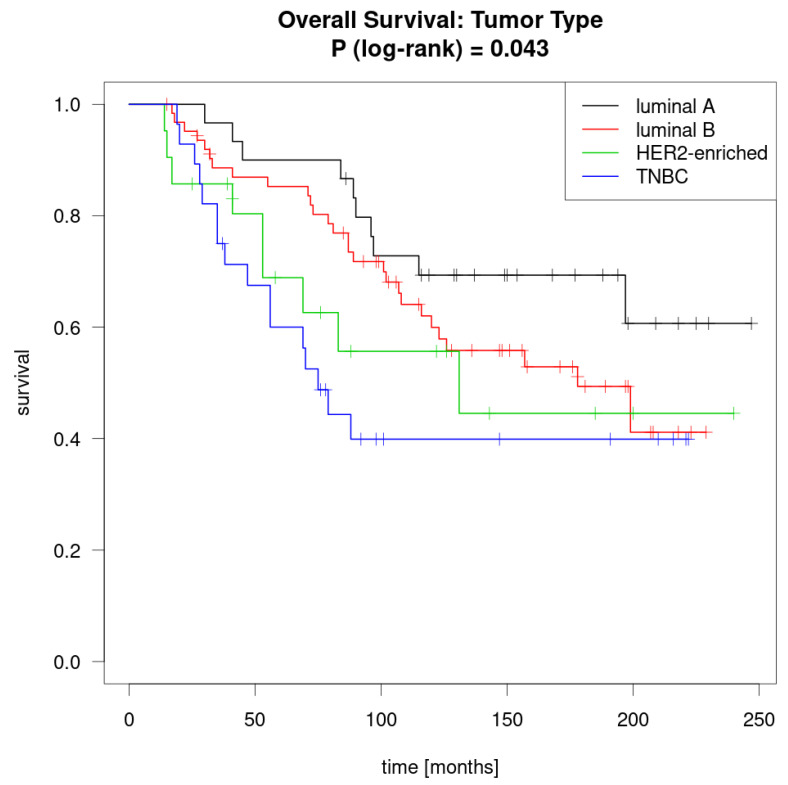
Kaplan-Meier curve for mean survival [months] by tumor subtype.

The mean survival rate was highest within the luminal-A-like primary tumor group (190.5 months), followed by the luminal-B-like group (154.8 months), then the HER2-enriched group (142.0 months), and finally the TNBC group (118.2 months). This was significant in the log rank test (*p* = 0.043) (Figure 3).

#### 3.1.9. Survival as a Function of Local Distance

We determined a threshold for local distance by creating two groups using maximally selected rank statistics determined by log-rank tests. The optimal threshold value was found to be 161.56 pixels. The five-year survival rate for the first group (≤162 pixels) was 70%, whereas the second group (>162 pixels) was 38%. The Monte Carlo simulated global *p* value for the maxstat test was *p* = 0.043. The Kaplan–Meier curves are presented in Figure 3. 

For the cox regression, stepwise variable selection was performed and the categorical distance group determined in the previous analysis (≤162 vs. >162 pixels), PR, tumor size, and time to recurrence were selected. Distance was a significant factor with HR = 1.83 and a *p*-value of 0.034, as were a positive PR status (HR = 0.47, *p* = 0.007), tumor size T3/4 (HR = 3.804, *p* < 0.001), and time to recurrence in years (HR = 0.867, *p* = 0.021) (Table 5). In a similar model with distance in pixels as a metric variable instead of a categorical distance grouping factor, distance was not significant.

## 4. Discussion

In this study, 142 patients with breast cancer were reviewed, who subsequently developed IBRT. By analyzing primary tumor and recurrent tumor characteristics and local distance between primary and recurrent breast cancer, the objective was to find correlation between changes in tumor characteristics as a function of local distance.

As with metastases, biomarker characteristics in IBTR change over time. PR changes at a higher frequency than ER, both from negative to positive and vice versa. These results are similar to those from other studies. The findings regarding time to recurrence by subtype and survival by subtype (with luminal-A-like tumors having the best prognosis and TNBC having the worst prognosis) are consistent with the literature and are not surprising [3,14,15,16].

In addition, the analysis of survival by nodal status and tumor size are in accordance with other work [3,22,23,24,25], and reflects clinical experience.

Recurrences of smaller tumors (T0-2) occur significantly closer to the primary site than those of larger tumors. A possible explanation could be the area covered by boost radiation. Unfortunately, we have no data about the radiation fields, therefore we could not further evaluate this.

Interestingly, no significant difference in local distance between the primary site and recurrence could be shown in the different subtypes, either by subtype of primary tumor or of recurrence.

Regarding the Cox model, a positive PR-status (HR = 0.470), larger tumors (HR = 3.804) and time to recurrence (HR = 0.867) are in accordance with known risk factors and are not surprising [20].

In our Cox model, local distance is a significant factor in survival (HR = 1.825). As there is no significant change in biomarkers regarding distance, the distance itself seems to play an important role. Most likely, a substantial number of recurrences occurred in the axillary pit (Figure 1b,e) and were characterized as IBTR following the given definition. It is known that recurrences in the axillary lymph nodes have a worse prognosis than recurrences in the breast [20]. Moreover, the distance to a recurrence in the axillary pit is often larger than the distance to a recurrence in the breast. This might be an explanation as to why a larger distance between the primary tumor and IBTR has a worse prognosis. However, as the cutpoint was selected by optimization, the Cox model may not be entirely accurate. It should therefore only serve for generating hypotheses, and should be further evaluated in future studies.

In the Kaplan–Meier analysis, on the other hand, the reported significant *p*-value regarding survival differences in groups defined by the optimal threshold for distance takes this optimization into account and is valid.

This is one of the first descriptions of survival relating to the distance between the primary tumor site and the recurrence site. The aim of this study was to examine the change in receptor status between the primary tumor and IBTR. The changes in ER and PR were in accordance with the literature and clinical experience. No significant differences were seen with regard to local distance for ER, PR, or HER2 status.

One study [3] showed a significantly better prognosis in the group in which the recurrence occurred elsewhere in the breast compared to that in which the recurrence occurred at the same site. Another study evaluating the location did not find any significant differences if the IBTR was within 3 cm of the primary tumor or not [20].

Our results regarding survival and local distance are in contrast to those results. However, in the first study, the patients were all treated before 1984, and in 64% of all cases, the ER status was unknown; furthermore, the treatment of breast cancer in general changed, so these results cannot really be compared. In the second study, the location was determined as the relative position to the index tumor by clinical information or mammographic reports. In contrast to our study, where the location was determined by mammograms and ultrasound pictures, the evaluation might be more precise.

One limitation of our study is the rather small sample size, but a long follow-up of 158 months makes the dataset valuable.

The lack of an absolute distance is a problem, as we only have a relative distance on a standardized breast. Therefore, the exact breast size and distance was not taken into account. The relative size of the breast in the pictogram can serve as a reference. For example, the total width of the upper body in the pictogram was 960 pixels, so 160 pixels corresponds to about 1/6 of the total width.

Altogether, the changes in biomarkers between the primary tumor and IBTR are relevant for clinical practice, and therefore the immunohistochemical profile has to be evaluated in cases of breast cancer recurrence. Not only should the changes in biomarkers influence the treatment of IBTR, but also the local distance of the recurrence should be evaluated, to clarify the overall risk to the patient.

## Figures and Tables

**Table 1:** Patients and tumor characteristics.

	*n*	Mean/%
**Age at diagnosis [years]**	142	55.01
**Age at menarche [years]**	126	13.66
**Parity**	129	1.75
**BMI [kg/m^2^]**	133	25.78
**HRT use (ever)**		
No	106	74.6
Yes	22	15.5
**Menopausal status**		
premenopausal	36	25.3
perimenopausal	6	4.2
postmenopausal	84	59.2
unknown	16	11.2

**Table 2:** Tumor characteristics.

			*n*	%
**Localization of recurrence**				
	Same quadrant as primary carcinoma		79	55.6
	Other quadrant as primary carcinoma		63	44.4
**Localization at primary diagnosis**				
	Left breast		81	57.1
		Upper outer	45	31.7
		Upper inner	18	12.7
		lower outer	6	4.2
		lower inner	12	8.5
	Right breast		61	42.9
		Upper outer	33	23.2
		Upper inner	16	11.3
		lower outer	5	3.5
		lower inner	7	4.9
**Localization at recurrence**				
	Left breast		81	57
		Upper outer	46	32.4
		Upper inner	18	12.7
		lower outer	9	6.3
		lower inner	8	5.6
	Right breast		61	43
		Upper outer	37	26.1
		Upper inner	13	9.2
		lower outer	6	4.2
		lower inner	5	3.5
**Grading**	1		12	8.5
	2		77	54.2
	3		46	32.4
	unknown		7	4.9
				
**tumor size (T)**	0		6	4.2
	1		66	46.5
	2		50	35.2
	3		5	3.5
	4		8	5.6
	missing		7	4.9
**nodal status (*n*)**	0		72	50.7
	1		56	39.4
	2		6	4.2
	3		1	0.7
	missing		7	4.9
**Tumor type at primary diagnosis**				
	Luminal-A-like		30	21.1
	Luminal-B-like		63	44.4
	HER2-enriched		21	14.8
	TNBC		28	19.7
**Tumor type at recurrence**				
	Luminal-A-like		31	21.8
	Luminal-B-like		52	36.6
	HER2-enriched		19	13.4
	TNBC		38	26.8
	unknown		2	1.4

**Table 3:** Recurrence characteristics with regard to subtype at primary diagnosis.

Subtype at Primary Diagnosis	Mean Distance between Primary and Recurrence Site [pixels] *	Mean Time to Recurrence [months] **
**Luminal-A-like (*n* = 30)**	124	55.27
**Luminal-B-like (*n* = 63)**	126	45.84
**HER2-enriched (*n* = 21)**	176	35.19
**TNBC (*n* = 28)**	137	29.61

* Kruskal–Wallis test: *p* = 0.449; ** Kruskal–Wallis test: *p* = 0.009; Benjamini–Hochberg corrected *p* = 0.038.

**Table 4:** Recurrence characteristics with regard to subtype at recurrence.

Subtype at Recurrence	Mean Distance between Primary and Recurrence Site [pixels] *	Mean Time to Recurrence [months] **
**Luminal-A-like (*n* = 31)**	86	56.5
**Luminal-B-like (*n* = 52)**	144	47.1
**HER2-enriched (*n* = 19)**	201	36.95
**TNBC (*n* = 38)**	138	33.16
**Missing (*n* = 2)**	*n*/a	*n*/a

* Kruskal–Wallis test: *p* = 0.067 ** Kruskal–Wallis test: *p* = 0.011 (Benjamini–Hochberg corrected *p* = 0.038).

**Table 5:** Cox-regression model.

Variable	HR	95-%-CI	*p*-Value
Distance >162 pixels (ref: ≤162 pixels)	1.825	1.047–3.182	0.034
PR pos (ref: PR neg)	0.470	0.272–0.815	0.007
pT3/4 (ref: pT0/1/2)	3.804	1.795–8.060	<0.001
Time to recurrence (years)	0.867	0.768–0.979	0.021

HR: hazard ratio; CI: confidence interval; pos: positive; neg: negative.

## References

[1] Rauschecker H.H., Clarke M.J., Gatzemeier W., Recht A. (2001). Systemic therapy for treating locoregional recurrence in women with breast cancer. Cochrane Database Syst. Rev..

[2] Leitlinienprogramm Onkologie (Deutsche Krebsgesellschaft, Deutsche Krebshilfe, AWMF) (2020). S3-Leitlinie Früherkennung, Diagnose, Therapie und Nachsorge des Mammakarzinoms, Version 4.3, AWMF Registernummer: 032-045OL. http://www.leitlinienprogramm-onkologie.de/leitlinien/mammakarzinom/.

[3] Haffty B.G., Fischer D., Beinfield M., McKhann C. (1991). Prognosis following local recurrence in the conservatively treated breast cancer patient. Int. J. Radiat. Oncol..

[4] Katz A., Strom E.A., Buchholz T.A., Thames H.D., Smith C.D., Jhingran A., Hortobagyi G., Buzdar A.U., Theriault R., Singletary S.E. (2000). Locoregional recurrence patterns after mastectomy and Doxorubicin-based chemotherapy: Implications for postoperative irradiation. J. Clin. Oncol..

[5] Recht A., Gray R., Davidson N.E., Fowble B.L., Solin L.J., Cummings F.J., Falkson G., Falkson H.C., Iv S.G.T., Tormey D.C. (1999). Locoregional failure 10 years after mastectomy and adjuvant chemotherapy with or without Tamoxifen without irradiation: Experience of the Eastern cooperative oncology group. J. Clin. Oncol..

[6] Taghian A.G., Jeong J.-H., Mamounas E., Anderson S., Bryant J., Deutsch M., Wolmark N. (2004). Patterns of locoregional failure in patients with operable breast cancer treated by mastectomy and adjuvant chemotherapy with or without Tamoxifen and without radiotherapy: Results from five national surgical adjuvant breast and bowel project randomized clinical trials. J. Clin. Oncol..

[7] Wallgren A., Bonetti M., Gelber R., Goldhirsch A., Castiglione-Gertsch M., Holmberg S., Lindtner J., Thürlimann B., Fey M., Werner I. (2003). Risk factors for locoregional recurrence among breast cancer patients: Results from international breast cancer study group trials I through VII. J. Clin. Oncol..

[8] Komoike Y., Akiyama F., Iino Y., Ikeda T., Akashi-Tanaka S., Ohsumi S., Kusama M., Sano M., Shin E., Suemasu K. (2006). Ipsilateral breast tumor recurrence (IBTR) after breast-conserving treatment for early breast cancer: Risk factors and impact on distant metastases. Cancer.

[9] Aurilio G., Disalvatore D., Pruneri G., Bagnardi V., Viale G., Curigliano G., Adamoli L., Munzone E., Sciandivasci A., de Vita F. (2014). A meta-analysis of oestrogen receptor, progesterone receptor and human epidermal growth factor receptor 2 discordance between primary breast cancer and metastases. Eur. J. Cancer.

[10] Lee J.H., Lee S.K., Park S.M., Ryu J.M., Paik H.J., Yi H.W., Bae S., Lee J.E., Kim S.W., Nam S.J. (2015). Independent prognostic factors for overall survival after salvage operation for ipsilateral breast tumor recurrence following breast-conserving surgery. J. Breast Cancer.

[11] Lowery A.J., Kell M.R., Glynn R.W., Kerin M.J., Sweeney K.J. (2012). Locoregional recurrence after breast cancer surgery: A systematic review by receptor phenotype. Breast Cancer Res. Treat..

[12] Schneeweiss A., Hartkopf A.D., Müller V., Wöckel A., Lux M.P., Janni W., Ettl J., Belleville E., Huober J., Thill M. (2020). Update breast cancer 2020 part 1—Early breast cancer: Consolidation of knowledge about known therapies. Geburtshilfe Frauenheilkd..

[13] Wöckel A., Festl J., Stüber T., Brust K., Krockenberger M., Heuschmann P.U., Jírů-Hillmann S., Albert U.-S., Budach W., Follmann M. (2018). Interdisciplinary screening, diagnosis, therapy and follow-up of breast cancer. Guideline of the DGGG and the DKG (S3-level, AWMF registry number 032/045OL, december 2017)—Part 2 with recommendations for the therapy of primary, recurrent and advanced breast cancer. Geburtshilfe Frauenheilkd..

[14] Grischke E.-M., Wallwiener D., Souchon R., Fehm T., Loehberg C.R., Jud S.M., Lux M.P., Beckmann M.W., Renner S.P. (2013). Isolated loco-regional recurrence of breast cancer—established and innovative therapy concepts. Geburtshilfe Frauenheilkd..

[15] Loehberg C.R., Almstedt K., Jud S.M., Haeberle L., Fasching P.A., Hack C.C., Lux M.P., Thiel F.C., Schrauder M.G., Brunner M. (2013). Prognostic relevance of Ki-67 in the primary tumor for survival after a diagnosis of distant metastasis. Breast Cancer Res. Treat..

[16] Okumura Y., Nishimura R., Nakatsukasa K., Yoshida A., Masuda N., Tanabe M., Shien T., Tanaka S., Arima N., Komoike Y. (2015). Change in estrogen receptor, HER2, and Ki-67 status between primary breast cancer and ipsilateral breast cancer tumor recurrence. Eur. J. Surg. Oncol..

[17] Jwa E., Shin K.H., Kim J.Y., Park Y.H., Jung S.Y., Lee E.S., Park I.H., Lee K.S., Ro J., Kim Y.J. (2016). Locoregional recurrence by tumor biology in breast cancer patients after preoperative chemotherapy and breast conservation treatment. Cancer Res. Treat..

[18] Wapnir I., Aebi S., Gelber S., Anderson S.A., Láng I., Robidoux A., Mamounas E.P., Wolmark N. (2008). Progress on BIG 1-02/IBCSG 27-02/NSABP B-37, a prospective randomized trial evaluating chemotherapy after local therapy for isolated locoregional recurrences of breast cancer. Ann. Surg. Oncol..

[19] Huang E., Buchholz T.A., Meric F., Krishnamurthy S., Mirza N.Q., Ames F.C., Feig B.W., Kuerer H.M., Ross M.I., Singletary S.E. (2002). Classifying local disease recurrences after breast conservation therapy based on location and histology: New primary tumors have more favorable outcomes than true local disease recurrences. Cancer.

[20] Panet-Raymond V., Truong P.T., Alexander C., Lesperance M., McDonald R.E., Watson P.H. (2010). Clinicopathologic factors of the recurrent tumor predict outcome in patients with ipsilateral breast tumor recurrence. Cancer.

[21] Aebi S., Gelber S., Anderson S.J., Lang I., Robidoux A., Martín M., Nortier J.W.R., Paterson A.H.G., Rimawi M., Baena-Cañada J.M. (2014). Chemotherapy for isolated locoregional recurrence of breast cancer (CALOR): A randomised trial. Lancet Oncol..

[22] Fowble B., Solin L.J., Schultz D.J., Rubenstein J., Goodman R.L. (1990). Breast recurrence following conservative surgery and radiation: Patterns of failure, prognosis, and pathologic findings from mastectomy specimens with implications for treatment. Int. J. Radiat. Oncol..

[23] Kurtz J.M., Amalric R., Brandone H., Ayme Y., Jacquemier J., Pietra J.C., Hans D., Pollet J.F., Bressac C., Spitalier J.M. (1989). Local recurrence after breast-conserving surgery and radiotherapy. Frequency, time course, and prognosis. Cancer.

[24] Touboul E., Buffat L., Belkacémi Y., Lefranc J.-P., Uzan S., Lhuillier P., Faivre C., Huart J., Lotz J.-P., Antoine M. (1999). Local recurrences and distant metastases after breast-conserving surgery and radiation therapy for early breast cancer. Int. J. Radiat. Oncol..

[25] Veronesi U., Marubini E., Del Vecchio M., Manzari A., Andreola S., Greco M., Luini A., Merson M., Saccozzi R., Rilke F. (1995). Local Recurrences and Distant Metastases After Conservative Breast Cancer Treatments: Partly Independent Events. J. Natl. Cancer Inst..

[26] R Core Team (2020). R: A Language and Environment for Statistical Computing. http://www.r-project.org/index.html.

